# The Effect of Game Playing and Goal Orientation on Creativity

**DOI:** 10.3389/fpsyg.2022.899694

**Published:** 2022-09-02

**Authors:** Jungim Mun

**Affiliations:** Department of Marketing, University of Massachusetts Boston, Boston, MA, United States

**Keywords:** creativity, new product development, cognitive game, fun, goal orientation

## Abstract

In an effort to bolster employee creativity, companies like Google and Groupon have adopted indoor work spaces that incorporate slides, swings, and unconventional design. While it may be costly and time-consuming to change certain aspects of a firm's work environment (e.g., the room design) to aid creativity and brainstorming, it is relatively easy for managers to encourage employees to engage in certain forms of unstructured recreation immediately prior to creative-based tasks for a new product development. This research addresses an important oversight in the literature by exploring the effect of cognitive game playing and goal orientation on subsequent new product development creativity. It was found that a cognitive game that engenders a greater degree of fun results in greater creativity on a subsequent new product development task, compared with both a cognitive-based game that engenders less fun and a control group. Furthermore, it was found that, for a cognitive-based game that engenders a high degree of fun, individuals who are primed with a process goal orientation are more likely to be creative on a subsequent new product development task than those who are primed with an outcome goal orientation.

## Introduction

A plethora of academic studies have discussed the important downstream outcomes of creativity. Specifically, products that are original and useful are more likely to appeal to consumers (Cooper and Kleinschmidt, 1987). Dahl et al. (1999) supported this claim by demonstrating the mediating role of originality and usefulness of product design on customer appeal. A creative firm that provides original and useful products meets the needs of consumers by developing innovative and superior products in the market (Cooper, 1979; Deshpande et al., 1993). Griffin and Page (1996) showed that creativity for new products and marketing programs are strong determinants of new product success. Thus, creativity entails differentiation from one's competitors in the marketplace (Andrews and Smith, 1996). Furthermore, the accumulation of organizational intelligence regarding original and useful ideas gives a firm a competitive advantage, which in turn results in a greater likelihood of new product success (Barney, 1991; Hunt and Morgan, 1995). Similarly, Dahl and Moreau (2002) suggested that consumers are willing to pay a higher price for more original product concepts. Hence, firms can benefit financially from increases in the originality of their new product.

The potential of game playing to facilitate creativity has recently begun to receive attention from creativity researchers. Prior research has examined the positive effect of motor skill games, role playing games, and videogames on creativity (Squire, 2006; Williams et al., 2006; Hamlen, 2009; Hutton and Sundar, 2010; Cavallera et al., 2011; Jackson et al., 2011; Lupu, 2012; Chung, 2013; Balance-Herrera et al., 2019). Cognitive stimulation and physiological arousal have accounted for the positive link between game playing and creativity. However, to the best of my knowledge, prior research has neither considered the potential underlying link between the level of *fun* experienced while playing a game and the subsequent creative outcomes nor examined the link between traditional cognitive-based games (e.g., math and pattern recognition games, brain teaser games, and card games) and creativity.

**Figure 1:**
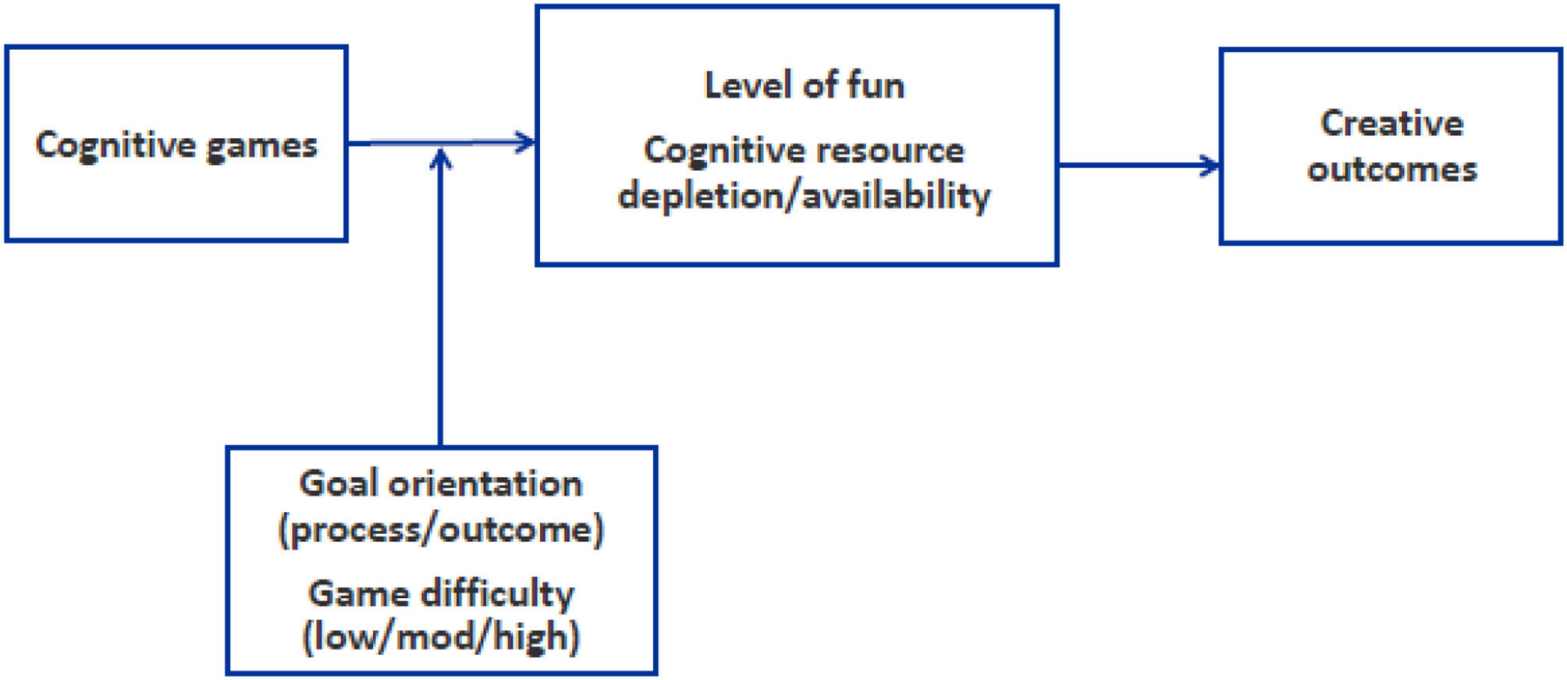
Overview of the theoretical model.

Hence, the first research question that I aim to answer is whether playing a cognitive-based game will enhance creativity and, if so, what is the key mechanism involved? To this end, I look at the centrality of fun as a process account in this highly “cognitive” domain. The second research question I attempt to answer is whether the effect of cognitive-based games on creativity, if any, depends on the goal orientation associated with the game itself (outcome-focused vs. process-focused). In Figure 1, an overview of the theoretical model underlying this research is shown.

## Theoretical Framework and Hypotheses

### Cognitive Games and Creativity: The Centrality of Fun in an Exclusively “Cognitive” Arena

Prior research has shown that game playing can positively influence subsequent creative outcomes (Squire, 2006; Williams et al., 2006; Hamlen, 2009; Hutton and Sundar, 2010; Cavallera et al., 2011; Jackson et al., 2011; Lupu, 2012; Chung, 2013). As previously mentioned, video games (Hamlen, 2009; Hutton and Sundar, 2010; Jackson et al., 2011; Balance-Herrera et al., 2019) have been shown to increase creativity. In the domain of children's education, it has been suggested that video games embody good learning principles and enable the development of both creativity and critical thinking skills (Squire, 2006).

According to Kinrade et al. (2010), games can be classified as either “primarily” cognitive (e.g., math and pattern recognition games, brain teaser games, and card games) or “primarily” motor skill—gross and/or fine—(e.g., golf-putting and ping-pong) in origin. Furthermore, according to Baumeister et al. (1990), some games are combination-based in that they are neither “primarily” cognitive nor motor skill in origin but rather require a mix of both cognitive decision-making and physical reactions. For example, with respect to the latter category, a video game (e.g., a joystick controlling a car) typically tests both cognitive and motor skills as it involves choice, reactions, hand control, and visual tracking.

Underlying process accounts of the above-referenced relationship between game playing and creativity have been couched in terms of physiological arousal (e.g., *Dance* and *Dance* video game) (Hutton and Sundar, 2010). Recently, Frith et al. (2020) also found that embodied movement robustly enhances creativity. In addition, although not a game *per se*, Benedek et al. (2012) showed that cognitive stimulation, due to engagement in a task that required individuals to generate random sequences of key responses, resulted in a greater number of ideas being generated on a subsequent task. However, I posit that the level of fun experienced in the course of a game playing experience should also play a central role in the relationship between game playing (even a cognitive one) and subsequent creativity.

Positive affect has been shown to enhance creative cognition (Isen et al., 1985, 1987; Fredrickson, 2001; Isen, 2001; Benedek et al., 2020). Similarly, Pillay et al. (2020) found that positive emotion elicited greater enthusiasm and confidence, which led to an increase in the quantity of new ideas. Moreover, Benedek et al. (2020) found that enjoyment compared with other motives such as social recognition and duty motives was the strongest motivation to driving everyday creativity. Fun is an experiential stimulation (Friedman et al., 2007) that engenders enjoyment and pleasure that people experience while they are engaged in particular actions. Importantly, fun is related to a specific type of positive affect resulting from engagement in certain experiences (Truhon, 1983; Mainemelis and Ronson, 2006; Pryor et al., 2010; Vijay and Vazirani, 2011).

Therefore, I predict that a cognitive game that engenders the greatest degree of fun will likewise lead to the greatest creative outcomes. Formally stated:

**H1:** A cognitive-based game that engenders a greater degree of fun will result in greater creativity on a subsequent new product development task, compared with a cognitive-based game that engenders less fun.
**H2:** A cognitive-based game that engenders a high degree of fun will result in greater creativity on a subsequent new product development task, compared with a control group.

### The Moderating Role of Goal Orientation

A considerable body of research has shown that the locus of motivation (i.e., intrinsic/extrinsic motivation) influences creative outcomes, and there is a consensus that intrinsic motivation enhances creativity relative to extrinsic motivation (Amabile, 1996; Deci et al., 1999). With respect to intrinsic motivation, individuals tend to focus on the process itself including personal feelings of interest, positive emotion, or competence. In contrast, with respect to extrinsic motivation, individuals tend to focus on the outcome including obtaining goals, rewards, or recognition (Fogeard and Mecklenburg, 2013). Consistently, Bakker et al. (2020) found that learning goal (vs. performance goal) orientation increased creativity in the workplace. Because creativity is an intellectual thought process requiring a great deal of cognitive effort (Simonton, 1977), the degree to which individuals are inherently interested in the problem and motivated to find a solution is necessary to be creative (Shalley and Oldham, 1985).

Previous research has shown that individuals in a positive mood state are more creative when working toward process-focused goals (i.e., enjoyment) and less creative when focusing on outcome-focused goals (i.e., performance) (Hirt et al., 1996). Importantly, fun is a specific type of positive affect resulting from engagement in certain experiences (Truhon, 1983; Mainemelis and Ronson, 2006; Pryor et al., 2010; Vijay and Vazirani, 2011). Hence, I hypothesize that when engaged in a cognitive-based game that engenders a high degree of fun, individuals will generally be in happy/positive mood states, and thus, a process-focused goal orientation with respect to the game should lead to greater creativity on a subsequent new product development task (vs. an outcome-focused goal orientation with respect to the game). Formally stated:

**H3**: Individuals who are primed with a process goal orientation for a cognitive-based game that engenders a high degree of fun will be more creative on a subsequent new product development task than those who are primed with an outcome goal orientation.

## Experiment 1

The purpose of experiment 1 is to test H1 and H2 to ascertain any cognitive game playing effects on subsequent creativity for a simple new product development task. A one-way ANOVA design was utilized.

### Procedure

In total, 147 undergraduates in a large state university participated in the experiment. All students were majoring in business administration. The average age of subjects was about 20.8 years. The sample includes 86 male students (58.5%) and 61 female students (41.5%). The following three games were utilized: (1) a symbolic-pattern recognition game (shape Sudoku) called *Genie-ous*; (2) a Rubik's cube, which involved spatial reasoning; and (3) an unrelated experimental wild card in the form of a combination cognitive-motor skill free-form drawing game^1^. Both *Genie-ous* and the Rubik's cube are employed in this study as treatment effects where participants use the cognitive domain but not motor skills or reaction. Since the Rubik's cube requires a certain level of spatial awareness and determination, it is considered to be less fun than *Genie-ous* for participants. Upon arrival, participants were randomly assigned to one of four conditions. Participants in the three game conditions were asked to engage in the given tasks first and then were asked to participate in a new product development task. The average time that participants engaged in the game tasks was 15–20 min (across three games)^2^. Participants in the control group did not engage in the game tasks. For the simple new product development task, all participants were asked to imagine themselves as a mattress manufacturer looking for creative ideas for a new kind of mattress (Mehta et al., 2012). Specifically, they were asked to read the following scenario:

“Please imagine yourself as a mattress manufacturer. Generate as many creative ideas for a new mattress. It may include a new feature or a new product as well.”

**Figure 2:**
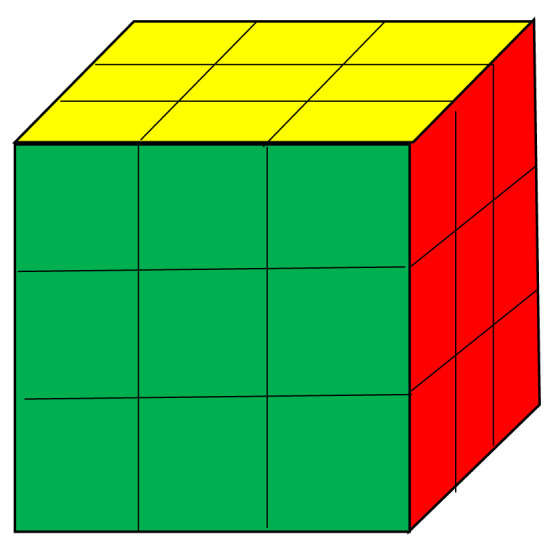
Rubik's cube game.

**Figure 3:**
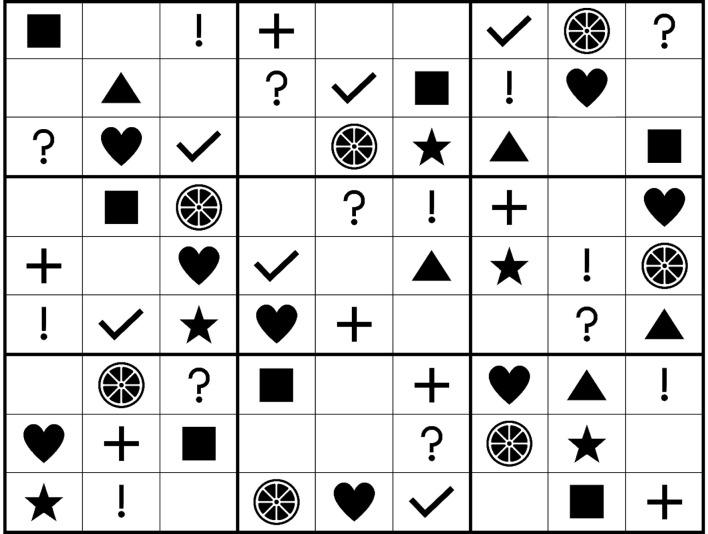
*Genie-ous* (shape sudoku) game.

After reading the imaginary scenario, participants were asked to generate as many creative ideas as possible for a new feature or a new product and to type each idea into the computer. No time limit was imposed for the idea generation task. Finally, participants answered questions that assessed the level of fun related to playing the games. The experiment concluded with various demographic questions. The Rubik's cube and *Genie-ous* (shape-sudoku) used in experiment 1 are shown in Figures 2, 3, respectively.

### Results

#### Number of Ideas Generated

Table 1 presents the results of experiment 1. Note that five responses were excluded and will be excluded for further analysis because those participants did not generate any ideas. The results showed that the number of ideas generated in the *Genie-ous* group (M = 4.03, *p* > 0.1) was not significantly different from those in the control group (M = 3.73). However, pairwise comparisons showed that participants in the Rubik's cube group (M = 2.97, *p* < 0.05) generated fewer ideas than those in the control group. Moreover, participants in the *Genie-ous* group generated significantly more ideas than those in the Rubik's cube group (*p* < 0.01).

**Table 1:** Summary of experiment 1.

	**Number of shapes used**	**Novelty**	**Usefulness**	**Overall creativity**	**Level of fun for game**
(1) Control (*n* = 44)	3.73	4.88	6.05	5.47	N/A
(2) *Genie-ous* (*n* = 31)	4.03	5.83	6.48	6.16	5.44
(3) Rubik's cube (*n* = 34)	2.97	4.73	5.61	5.17	4.49
(4) Drawing (*n* = 33)	3.45	5.30	6.06	5.68	4.41
Total (*n* = 142)	3.55	5.15	6.04	5.59	4.78
***p*** **-Value**					
Diff: (1)-(2) [H2]	ns	**	ns	*	ns
Diff: (1)-(3)	**	ns	ns	ns	ns
Diff: (2)-(3) [H1]	***	**	*	**	***

***, **, and * denote significance at 1, 5, and 10% levels, respectively, from two-tailed. “ns” denotes a lack of statistical significance at the 10% level.

#### Creativity of the Ideas Generated

Two coders blind to the experimental conditions identified the most creative idea that each participant generated. The difference in selection was resolved through discussion between the coders. Each coder then rated the most creative idea generated by each participant. The correlation between the coders' ratings was positive (0.43) and significant (*p* < 0.01). This measure allowed us to assess creativity, independent of the number of ideas each participant generated (Yang et al., 2012). In other words, participants who generated one creative idea alongside many average ideas would not be penalized for their less creative ideas. Furthermore, those who generated one highly creative idea, but few additional ideas, would not be penalized either. Both creativity dimensions were measured (novelty and usefulness). Three items captured each of the two dimensions (Dahl et al., 1999; Moreau and Dahl, 2005; Burroughs et al., 2011).

For novelty, the three items were measured with a 7-point Likert scale (α = 0.98): 1 = not at all original/7 = very original; 1 = not at all innovative/7 = very innovative; 1 = not at all novel/7 = very novel. Pairwise comparisons showed that the ideas generated by participants in *Genie-ous* group (M = 5.83) were significantly more original than the ideas generated by participants in the control group (M = 4.88, *p* < 0.05) or by those in the Rubik's cube group (M = 4.73, *p* < 0.05).

For usefulness, three items were measured as well (α = 0.98): 1 = not at all useful/7 = very useful; 1 = not at all effective/7 = very effective; 1 = not at all worthwhile/7 = very worthwhile. Ideas generated by participants in the *Genie-ous* group (M = 6.48) were rated more useful than those in the Rubik's cube group (M = 5.61, *p* = 0.07).

In this research, I am more interested in overall creativity than in one of the two separate constructs, so I averaged the six items. Pairwise comparisons showed that the ideas generated by the *Genie-ous* group (M = 6.16) were more creative than those in the Rubik's cube group (M = 5.17, *p* < 0.05) and marginally more creative than the control group (M = 5.47, *p* = 0.08).

#### Fun

Participants were asked to rate the level of fun for the game in which they were engaged. Three items were measured with a 7-point Likert scale [modified from Carver and White (1994)]: 1 = not at all interesting/7 = very interesting; 1 = not at all exciting/7 = very exciting; 1 = not at all engrossing/7 = very engrossing (α = 0.87). The level of fun experienced in the *Genie-ous* group (M = 5.39) was significantly higher than in the Rubik's cube group (M = 4.49, *p* < 0.01). Further results of *t*-tests for the level of fun found that only the *Genie-ous* group (*t* = 8.16, *p* < 0.01) was significantly higher than the mid-point for the level of fun. The level of fun for the Rubik's cube group (*t* = 1.95, *p* = 0.060) was only marginally higher than the mid-point for the level of fun.

#### Mediation Analyses

Mediation analysis was conducted in order to test whether the level of fun is responsible for the effect of game playing on creative outcomes. Following the study by Preacher and Hayes (2004), a bootstrapping approach using 5,000 bootstrapping samples was used to assess the mediation effect. The result demonstrated that the 95% confidence interval for the Rubik's cube group and the *Genie-ous* group contrast, for the number of ideas (−0.73– −0.06), usefulness (−0.96– −0.02), and overall creativity (−0.73– −0.03), did not include zero, which suggests that an indirect mediation effect for the level of fun was present. For idea originality (−0.67–0.06), it did include zero, which indicates a lack of a mediating role for fun in this regard.

### Discussion

Experiment 1 showed that a cognitive game that engendered a greater level of fun resulted in more creative outcomes on a subsequent new product development task, compared with a cognitive game that did not engender as much fun. In addition, a cognitive game that engendered a high degree of fun resulted in greater creativity on a subsequent new product development task, compared with a control group.

These findings in experiment 1 have important implications. While prior research investigates the effect of playing games on subsequent creative outcomes, it mainly focuses on video games in which subjects are allowed to use both cognition and motor skills (Squire, 2006; Hamlen, 2009; Hutton and Sundar, 2010; Jackson et al., 2011; Balance-Herrera et al., 2019). Meanwhile, other studies focus on how sports or physical activity affect creativity (Cavallera et al., 2011; Lupu, 2012). In contrast, by using *Genie-ous* (a shape Sudoku) which is a cognitive-based game, I am able to isolate the effect of cognition-based fun on creativity from that of motor skills or physiological activity. Thus, this study adds to the literature by demonstrating the uniqueness of cognition-based fun as a process accounting for the link between game playing and subsequent creativity.

### Post-test

The purpose of the post-test was to test the alternative explanation that differential levels of cognitive stimulation could possibly account for the pattern of results found in experiment 1. Hence, the post-test was designed to measure cognitive stimulation levels for the two cognitive-based games (*Genie-ous* and Rubik's cube) I adopted in experiment 1.

#### Procedure

As I attempted to test different levels of cognitive stimulation induced by cognitive-based games, a control group was not included in the post-test. Thus, the post-test consisted of a one-way ANOVA design. It included the Rubik's cube and the *Genie-ous* condition. Forty-two undergraduates (male = 26, average age = 20.80) in a large state university participated in the post-test in exchange for class credit. Upon arrival, they were randomly assigned to one of two groups. After performing the given game, participants were asked to rate the cognitive stimulation level for the given task on a 9-point Likert scale (1 = highly disagree, 9 = highly agree). The scale consisted of the following questions: “When I was engaged in the task, I felt that my thinking process was actively stimulated; the task encouraged me to think intellectually; the task facilitated my intellectual functioning [α = 0.71, modified from Bolkan and Goodboy (2010)].” Finally, participants answered demographic questions and debriefed.

#### Results

One-way ANOVA was conducted to test cognitive stimulation between the two games. It was found that cognitive stimulation was not significantly different across the two games [*F* (1, 40) = 1.57, *p* > 0.1]. Cognitive stimulation for the Rubik's cube was 6.79 (*n* = 21), and for the *Genie-ous*, it was 7.24 (*n* = 21). Thus, I could eliminate the possibility of the alternative explanation that differential levels of cognitive stimulation used between the two games accounted for the pattern of results found in experiment 1.

#### Discussion

Post-test showed that cognitive stimulation levels for the two cognitive-based games (*Genie-ous*, and the Rubik's cube) in experiment 1 were not significantly different. Hence, cognitive stimulation did not account for the pattern of results in experiment 1. By holding the level of cognition constant, the post-test results enhance our understanding of the central role of fun in creative outcomes for a new product development task, as discussed by Baumeister et al. (1990) and Kinrade et al. (2010).

## Experiment 2

**Figure 4:**
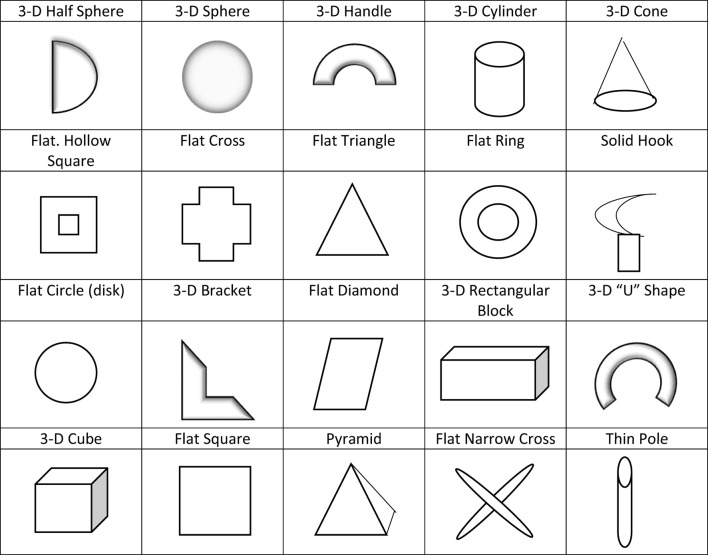
Twenty shapes for toy idea in experiment 2. Twenty shapes shown in this figure can serve as components for your toy idea (Moreau and Dahl, 2005; Yang et al., 2012).

Experiment 2 was designed to test H_3_, which adopts one-way ANCOVA (goal orientation: process vs. outcome). The purpose was to examine the effect of a goal orientation context on the subsequent link between cognitive-based game playing and creativity. Goal orientation was manipulated by giving specific (process vs. outcome goal orientation) instructions for the given pattern recognition game (shape Sudoku). For the subsequent new product development task, designing a toy for a child was used (Moreau and Dahl, 2005). The dependent variables included two constructs of creativity (novelty and usefulness). Mood and self-efficacy were measured as covariates; 20 shapes for toy ideas used in experiment 2 are shown in Figure 4.

### Procedure

A total of 81 undergraduate students (male = 40, average age = 21.1) from a large state university participated in this experiment in exchange for class credit. Upon arrival, participants were randomly assigned to one of two groups. Depending on the group to which they were assigned, participants read the goal orientation instructions related to the pattern recognition game (shape Sudoku) for the goal orientation manipulation. Participants in the process goal orientation condition read the instruction for the given task (focus on the process of playing the game), whereas participants in the outcome goal orientation condition read another instruction (focus on the outcome of winning). The specific instructions for goal manipulation were as follows.

*Process-focused goal orientation [modified from Escalas and Luce (**2003**)]*: The following game is provided for you to play. Please focus on the process of playing the game. The primary goal is not speed and/or how many boxes you can fill in. Final scores will not be computed and distributed.

*Outcome-focused goal orientation [modified from Burroughs et al. (**2011**)]*: The following game is provided for you to play. Please focus on the outcome of winning. The primary goal is speed and/or how many boxes you can fill in. Final scores will be computed and distributed.

After completing the pattern recognition game, participants were asked to complete the subsequent new product development creativity task. The new product development task of designing a toy for children was used. Participants read the instruction “Please design a toy, anything a child of age 5–11 can use to play with. Twenty shapes can serve as components for your toy idea” (Moreau and Dahl, 2005; Yang et al., 2012; see Figure 1).

Fun was measured in order to test the expected process mechanism for goal orientation. In the same vein as in experiment 1, the three items used to measure fun included “I found the new product design task; interesting, exciting, and engrossing”; 1 = highly disagree, 7 = highly agree [modified from Carver and White (1994)].

Participants then rated covariates, mood, and self-efficacy. To measure mood, the PANAS scale of 20 items with a 5-point Likert scale was used (Watson et al., 1988). The 10 items indicating a positive mood were as follows: interested, excited, strong, enthusiastic, proud, alert, inspired, determined, attentive, and active. Meanwhile, the 10 items indicating a negative mood were as follows: distressed, upset, guilty, scared, hostile, irritable, ashamed, nervous, jittery, and afraid. The construct of perceived self-efficacy reflects an optimistic self-belief (Jerusalem and Schwarzer, 1992). This is the belief that one can perform novel or difficult tasks, or cope with adversity in various domains of human functioning. Perceived self-efficacy facilitates goal-setting, effort investment, persistence in the face of barriers, and recovery from setbacks. Therefore, it can be regarded as a positive resistance resource factor. Ten items with a 4-point Likert scale were used to measure perceived self-efficacy including “I can always manage to solve difficult problems if I try hard enough,” “If someone opposes me, I can find the means and ways to get what I want,” and “It is easy for me to stick to my aims and accomplish my goals” (1 = not at all true; 4 = exactly true).

Finally, manipulation check questions for goal orientation, a suspicion probe, and demographic questions were asked. The average time that participants engaged in the experiment was 15–20 min.

### Results

#### Number of Components Used for Toy Design

Table 2 presents the results of experiment 2. The participants used 5.66 components out of 20 shapes when designing a toy (SD = 5.21). One-way ANCOVA found that goal orientation (M_process_ = 5.51, M_outcome_ = 5.82) did not have a significant effect on the number of components used for toy design [*F* (1, 75) = 0.78), *p* > 0.1]. Among the covariates of mood and self-efficacy, only positive affect had a significant effect on this measure [F (1, 75) =4.27, *p* < 0.05]. It was further found that participants tended to use more components when they felt a more positive affect (*r* = 0.24, *p* < 0.05). Negative affect [F (1, 75) < 0.1, *p* > 0.1] and self-efficacy [F (1, 75) = 0.78, *p* > 0.1), by contrast, were not significant at all.

**Table 2:** Summary of experiment 2.

	**Number of shapes used**	**Novelty**	**Usefulness**	**Overall creativity**	**Level of fun for game**
(1) Process (*n* = 39)	5.51	4.00	4.00	4.00	4.75
(2) Outcome (*n* = 41)	5.82	3.23	3.27	3.25	3.88
Total (*n* = 80)	5.66	3.62	3.64	3.63	4.33
***p*-Value**					
Diff: (1)-(2) [H3]	ns	**	**	**	***
Covariate					
Positive effect	*	*	*	*	***
Negative effect	ns	ns	ns	ns	ns
Self-efficacy	ns	ns	ns	ns	ns

***, **, and * denote significance at 1, 5, and 10% levels, respectively, from two-tailed. “ns” denotes a lack of statistical significance at the 10% level.

#### Creativity of the Ideas Generated

**Figure 5:**
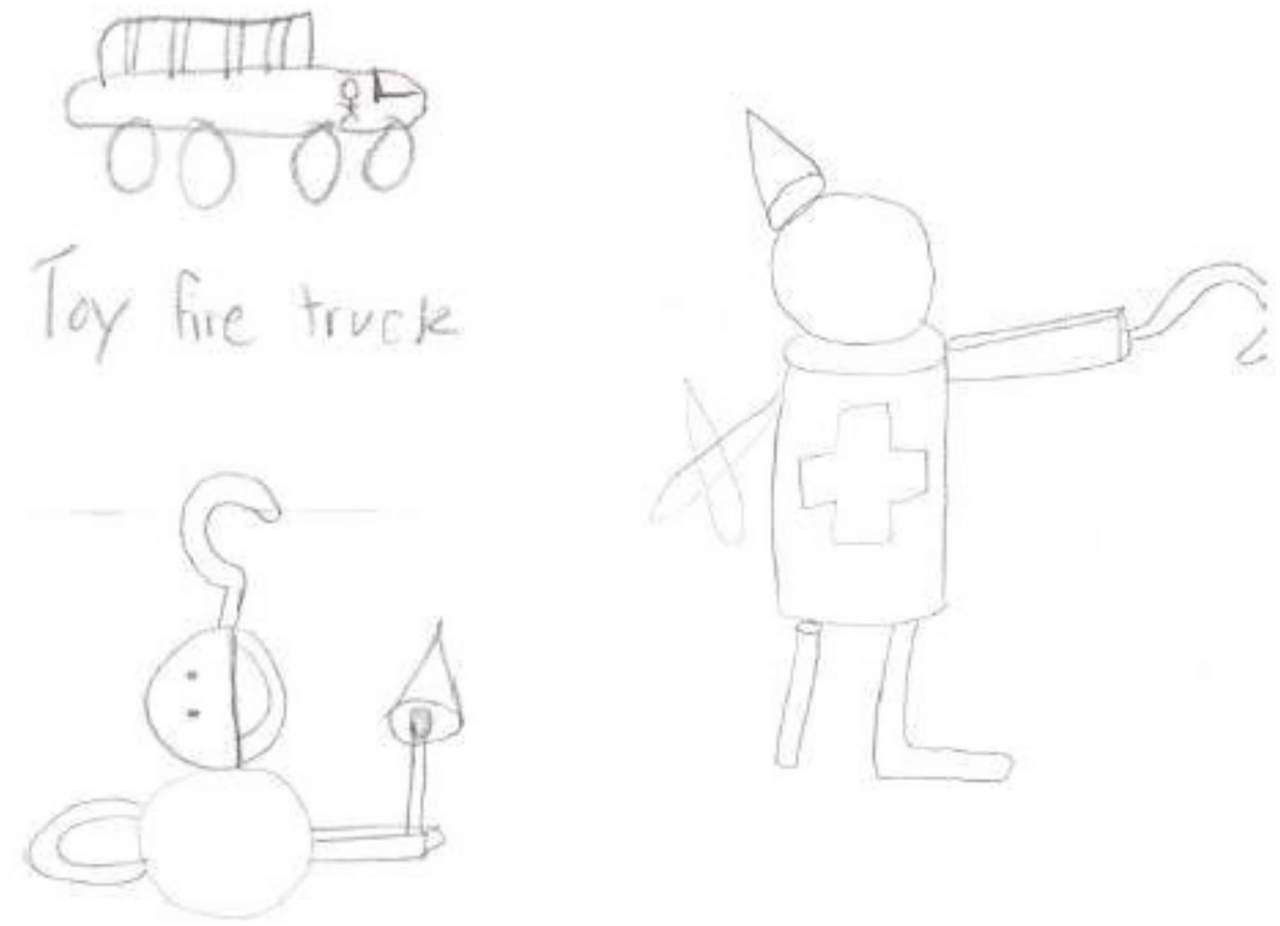
Examples of toy designs in experiment 2.

Examples of toys designed by participants are shown in Figure 5. Two independent coders blind to the experimental conditions rated the toy drawings of each participant according to six items of creativity, which consisted of two constructs, namely, novelty and usefulness. The correlation between the coders' ratings was positive (*r* = 0.75) and significant (*p* < 0.01). Just as in experiment 1, creativity was measured as two constructs, namely, novelty and usefulness. Three items captured each of the two dimensions (Dahl et al., 1999; Moreau and Dahl, 2005; Burroughs et al., 2011).

For the novelty dimension, three items were measured with a 7-point Likert scale (α = 0.98; original, innovative, novel). The novelty across two conditions was significant [F (1, 75) = 4.64, *p* < 0.05]. The toys designed by participants who were primed with a process goal orientation (M_process_ = 4.00) were significantly more original than the ideas generated by participants with an outcome goal orientation (M_outcome_ = 3.23). Among covariates, only positive affect had a marginal effect on this measure [F_positiveaffect_ (1, 75) =3.40, *p* < 0.1], but negative affect [F_negativeaffect_ (1, 75) = 2.71, *p* > 0.1] and self-efficacy [F_self−efficacy_ (1, 75) < 1, *p* > 0.1] were not significant.

For the usefulness dimension, three items were measured as well (α = 0.99; useful, effective, worthwhile). The usefulness across two conditions was significant [F (1, 75) = 4.15, *p* < 0.05]. Toys designed by participants in the process goal orientation group (M_process_ =4.00) were rated more useful than those in the outcome goal orientation group (M_outcome_ = 3.27). Again, only positive affect was found to be significant [F_positiveaffect_ (1, 75) = 4.05, *p* < 0.05] on this measure among covariates [F_negativeaffect_ (1, 75) = 2.31, *p* > 0.1; F_self−efficacy_ (1, 75) < 1, *p* > 0.01]. Participants tended to generate more useful ideas when they had a more positive affect (*r* = 0.20, *p* < 0.1).

Just as in experiment 1, I averaged the six items because the current research was more interested in overall creativity than in one of the two separate constructs. The results showed that overall creativity was significant [F (1, 75) = 4.54, *p* < 0.05]. The toy design generated by the process goal orientation group (M_process_ = 4.00) was more creative than the outcome goal orientation group (M_outcome_ =3.25). Also, the level of fun for *Genie-ous* (M = 4.33) was higher than the mid-point of the scale (*t* = 5.49, *p* < 0.01). Thus, it can be asserted that H_3_ was supported because individuals who were given a process goal orientation for a cognitive-based game that engendered a high degree of fun were more creative on a subsequent NPD task than those who were given an outcome goal orientation. In a similar vein to other measures, only positive affect was shown to be marginally significant [F (1, 75) = 3.84, *p* < 0.1] among covariates [F_negativeaffect_ (1, 75) =2.60, *p* > 0.1; F_self−efficacy_ (1, 75) <1, *p* > 0.01]. Moreover, toy designs were marginally more creative when participants felt a more positive affect (*r* = 0.19, *p* < 0.1).

#### Fun

Participants were asked to rate the level of fun for the given pattern recognition game (shape Sudoku) in which they were engaged. As was the case in experiment 1, three items were measured with a 7-point Likert scale (α = 0.93; interesting, exciting, engrossing). The level of fun across two goal orientation conditions was significant [F (1, 75) = 10.04, *p* < 0.01. The level of fun in the process goal orientation (M_process_= 4.75) was significantly higher than the outcome goal orientation group (M_outcome_= 3.88). Only positive affect was shown to be significant [F_positiveaffect_ (1, 75) = 9.94, *p* < 0.05] among covariates [F_negativeaffect_ (1, 75) < 1, *p* > 0.1; F_self−efficacy_ (1, 75) < 1, *p* > 0.1]. Furthermore, participants significantly rated the game as more fun when they felt more positive affect (*r* = 0.32, *p* < 0.05).

### Mediation Analyses

Mediation analysis was conducted in order to test whether fun was playing a mediating role in the relationship between goal orientations (process vs. outcome) and creative outcomes. Preacher and Hayes' (2004) bootstrapping approach was adopted, in which 5,000 bootstrapping samples were used to assess the mediation effect. The results showed that the 95% confidence interval for novelty (0.11–0.83), usefulness (0.09–0.76), and overall creativity (0.11–0.80) did not include zero, which suggested that an indirect mediation effect for the level of fun was present.

### Manipulation Check

A manipulation check was conducted to determine whether the manipulation for goal orientation (process vs. outcome) was successful. As intended, participants who were primed with a process goal orientation rated significantly higher for the questions “I was focused on playing for the sake of playing games [M_process_ = 5.49, M_outcome_ = 4.45, F (1, 79) =7.90, *p* < 0.01]” and “I was focused on playing for the challenging of playing games [M_process_ = 5.66, M_outcome_ = 4.93, F (1, 79) = 4.14, *p* < 0.05]” compared to those with an outcome goal orientation. Also, participants who were manipulated with an outcome goal orientation rated significantly higher for the questions “I was focused on playing in order to score points [M_outcome_ = 5.15, M_process_ = 3.24, F (1, 79) = 21.47, *p* < 0.01]” and “I was focused on playing in order to win [M_outcome_ = 5.40, M_process_ = 3.83, F (1, 79) = 12.13, *p* < 0.01]” compared to those with a process goal orientation. Hence, the manipulation of goal orientation was entirely successful in the current experiment.

### Discussion

The results from experiment 2 showed the effects of goal orientation (process vs. outcome) on creative outcomes in terms of novelty, usefulness, and overall creativity. More specifically, the toys designed by participants who were primed with a process goal orientation were significantly more original, useful, and overall creative than the toys designed by participants with an outcome goal orientation. Furthermore, the level of fun in the process goal orientation was significantly higher than that in the outcome goal orientation group. Among the covariates of mood and self-efficacy, only positive affect was shown to have a significant influence on the novelty, usefulness, overall creativity, and level of fun. Positive affect was found to be positively related to the usefulness dimension, overall creativity, and the level of fun as well. The mediating role of the level of fun was detected for each dimension of novelty and usefulness, as well as overall creativity. Moreover, as in the previous experiment, the level of fun for the given shape Sudoku was rated significantly higher than the mid-point scale.

Therefore, I found support for H_3_, which stated that individuals who were given a process goal orientation for a cognitive-based game that engendered a high degree of fun were more creative on a subsequent new product development task than those who were given an outcome goal orientation. Prior studies are muted about how the association between fun, which is a specific type of positive effect (Truhon, 1983; Mainemelis and Ronson, 2006; Pryor et al., 2010; Vijay and Vazirani, 2011), and creativity varies across the level of goal orientation. Therefore, the evidence documented in this section fills the gap in the literature on process goal orientation (Amabile, 1996; Deci et al., 1999) such as positive affect and creativity (Hirt et al., 1996).

## Contribution

This research addresses the effect of cognitive game playing on subsequent new product development creativity and the role of the degree of fun experienced as possible process accounts for the pattern of results derived.

Theoretically, this research seeks to expand the creativity literature by exploring the effect of cognitive game playing and goal orientation on creativity and by examining the centrality of fun in driving creative outcomes with respect to game playing, in general, and even in a “cognitive” domain.

The potentially substantive and managerial contributions of this research include the ability to provide managers with a timely and cost-effective lever for increasing creative outcomes. While it may be costly and time-consuming to change certain aspects of a firm's work environment (e.g., the room design) to aid creativity and brainstorming, it is relatively easy for managers to encourage employees to engage in certain forms of unstructured recreation such as games prior to brainstorming sessions for a new product development.

Similarly, organizations or educators may seek to develop in-house, unstructured employee recreation training programs that are focused in whole or part around the concept of utilizing game playing at work to enhance individual employee creativity on a day-to-day basis and with respect to a plethora of creativity-based work-place tasks.

This study is subjected to several caveats. I acknowledge that the participants were given unlimited time to play each game. Although this design was to ensure that they had the choice to stop playing each game when they had completed it or else lost interest in it, the average time engaged was not consistent across Genie-ous, Rubik's cube, and drawing. This may affect inferences made from the experiments. I also note that the number of subjects used in experiments is somewhat small. Caution is required in making inferences from the documented evidence. Finally, this study does not fully exploit the differential effects of fun activity on creative outcomes. For example, by playing video games, participants are able to use different levels of both cognition and motor skills. Future research that utilizes various video games to examine the effect of fun on creativity is warranted. Investigating not only the effect of cognitive stimulations but also the effect of emotional competencies on creativity seems promising.

## Data Availability Statement

The raw data supporting the conclusions of this article will be made available by the authors, without undue reservation.

## Ethics Statement

The studies involving human participants were reviewed and approved by State University of New York–Buffalo, Institutional Review Board (IRB). The patients/participants provided their written informed consent to participate in this study.

## Author Contributions

The author confirms being the sole contributor of this work and has approved it for publication.

## Conflict of Interest

The author declares that the research was conducted in the absence of any commercial or financial relationships that could be construed as a potential conflict of interest.

## Publisher's Note

All claims expressed in this article are solely those of the authors and do not necessarily represent those of their affiliated organizations, or those of the publisher, the editors and the reviewers. Any product that may be evaluated in this article, or claim that may be made by its manufacturer, is not guaranteed or endorsed by the publisher.

## Footnotes

^1^Not only is this a combination cognitive-motor skill game but its cognitive dimension is also different than what would be considered a traditional cognitive game (see Kinrade et al., 2010) in that it is “solution-less,” that is, it requires open-form versus closed-form cognition (such as a verbal, math, shape, or spatial puzzle/game).

^2^Subjects were given unlimited time to play each game in order to ensure that they had the choice to stop playing each game when they had completed it or else lost interest in it. The average time for Genie-ous, Rubik's cube, and drawing were ~18, 20, and 15 min, respectively. Applying caution is advised to readers as different times across games may affect the results.
